# Pharmacotherapy of restricted/repetitive behavior in autism spectrum disorder:a systematic review and meta-analysis

**DOI:** 10.1186/s12888-020-2477-9

**Published:** 2020-03-12

**Authors:** Yanjie Yu, Ashmita Chaulagain, Sindre Andre Pedersen, Stian Lydersen, Bennett L. Leventhal, Peter Szatmari, Branko Aleksic, Norio Ozaki, Norbert Skokauskas

**Affiliations:** 1 Department of Psychiatry, Nagoya University Graduate School of Medicine, Nagoya, Aichi Japan; 2 Regional Center for Child and Youth Mental Health and Child Welfare, Norwegian University of Science and Technology, Trondheim, Norway; 3 Library Section for Medicine and Health Science, Norwegian University of Science and Technology, Trondheim, Norway; 4 Division of Child & Adolescent Psychiatry, University of California, San Francisco, San Francisco, USA; 5 Department of Psychiatry, University of Toronto, Toronto, Ontario Canada

**Keywords:** Autism Spectrum disorder, Restricted/repetitive behavior, Pharmacotherapy, Systematic review, Meta -analysis

## Abstract

**Background:**

This paper is a systematic review and meta-analysis of the efficacy of available medications for the treatment of restricted/repetitive behavior (RRBs) in Autism Spectrum Disorder (ASD).

**Method:**

We searched MEDLINE, Embase, PsycINFO, The Cochrane Library (Cochrane Database of Systematic Reviews (CDRS), the Cochrane Central Register of Controlled Trials (CENTRAL), database of Abstracts of Reviews of Effects (DARE)), Scopus, Epistimonikos, Clinicaltrials.gov, and included all randomized controlled trials published after 1993 that were directed at RRBs in patients with ASD of all ages. We extracted the relevant data from the published studies with a predefined data extraction form and assessed the risk of bias. The primary outcomes were change in restricted/repetitive behavior. We performed a meta-analysis using the random effect model and included studies with given mean and standard deviation. This study is registered with PROSPERO number CRD42018092660).

**Results:**

We identified 14 randomized controlled trials that met initial inclusion criteria. After closer inspection, nine trials – involving 552 patients in total – were included in the final analysis. The meta-analysis found no significant difference between medications (including fluvoxamine, risperidone, fluoxetine, citalopram, oxytocin, N-Acetylcysteine, buspirone) and placebo in the treatment of RRBs in ASD (*P* = 0.20). Similarly, the sub-group meta-analysis also showed no significant difference between Selective Serotonin Reuptake Inhibitor (SSRIs) and placebo in the treatment of RRBs in ASD (*P* = 0.68). There was no evidence of publication bias.

**Conclusion:**

This meta-analysis finds little support for the routine use of medications to treat restricted/repetitive behaviors in Autism Spectrum Disorder. Further research of large, balanced trials with precise assessment tools and long-term follow-up are needed.

**Trial registration:**

The study protocol is registered in PROSPERO (Reference number: CRD42018092660).

## Background

Autism spectrum disorder (ASD) is a neurodevelopmental disorder characterized by early-onset, persistent deficits in social communication and social interaction and restricted, repetitive patterns of behavior, interests, or activities. The syndrome may also include deficits in verbal and nonverbal communication, and other behavioral and social symptoms [1, 2]. While there has been some controversy about the prevalence of ASD [3], prevalence estimates in the range of 2–3% of the population have been consistent [4–6]; similar prevalence has been reported in different countries with ASD apparently occurring in all racial, ethnic, and socioeconomic groups [7, 8].

Restricted and repetitive behavior (RRB) is a core feature of ASD [9, 10], which include repetitive motor phenomena (e.g., stereotypies), narrow or circumscribed interests, compulsions, and severe problematic behaviors, such as self-injury [11]. Clinically, RRB represent a major challenge for individuals with ASD and their families – including severe family distress and dysfunction due to the patients’ intolerance of change and acts of aggression against themselves or others [10, 12]. RRB’s appear to persist across development. While there are studies supporting non-medical treatment (i.e. Behavioral intervention, CBT) for reducing some types of RRB in individuals with ASD [12, 13], the pathophysiology and function of repetitive behaviors is still largely unresolved. RRB’s appear to persist across development [14].

The U.S. Food and Drug Administration (FDA) has approved two medications, risperidone and aripiprazole, for the pharmacological treatment of irritability associated with ASD [15]. However, no medications have been approved for the management of ASD core symptoms – social deficit and/ or RRBs [16]. A recent study indicate that 27.2% of youth with ASD receive psychotropic medications [17]. These medications are often not risperidone and aripiprazole and often fail to target core symptoms of the disorder [18, 19]. Some researchers report that individuals with ASD tends to respond less favorably to medications [20] and experience more frequent and severe adverse effects from medications [21]. In order for clinicians to make informed decisions for their patients, it is important to review the current evidence-base for the risks and benefits associated with ASD pharmacotherapy.

This paper reviews the evidence for using pharmacotherapy to treat RRBs in individuals with ASD based on available randomized controlled trials (RCTs).

## Methods

This meta-analysis followed the preferred reporting items for systematic reviews and meta-analysis (PRISMA) guidelines http://prisma-statement.org/prismastatement/Checklist.aspx [22]. A completed PRISMA checklist is available as Additional file 1.

### Protocol and registration

The study protocol is registered in PROSPERO (Reference number: CRD42018092660).

### Eligibility criteria

#### Study design

Only randomized controlled trials (RCTs) were eligible for inclusion in the meta-analysis.

#### Population

Patients of any age or gender were eligible if they had a DSM IV- or ICD10 diagnosis of autistic disorder (AD), or DSM 5 diagnosis of ASD, and measures of the restricted/repetitive or compulsive behaviors.

#### Intervention

Eligible studies utilized any pharmacologic intervention directed at the treatment of individuals with ASD who had restricted/repetitive behaviors or interests, including compulsive behaviors and stereotypies.

#### Comparison

Placebo.

#### Outcome

Relevant studies had to address changes in restricted/repetitive behavior or interests or compulsive behavior and stereotypies measured using: Repetitive Behavior Scale-Revised; Aberrant Behavior Checklist (ABC)/ (Stereotypic Behavior Subscale); Yale Brown Obsessive Compulsive Scale (Y-BOCs) and its child version (CY-BOCS).

#### Other

The focus on studies published in 1994, and beyond, was chosen since this was the publication year of the Diagnostic and Statistical Manual, Fourth Edition (DSM-IV) [23]. The manual established the evidence-based, standard criteria for the diagnosis of Autistic Disorder as one of the Pervasive Developmental Disorders. These are identical to the ICD-10 [24] criteria, allowing for diagnostic consistency across trials. Taken together, the DSM and ICD criteria are included in most standardized diagnostic instruments and they are generally consistent with DSM-5 [25] criteria for ASD.

Published studies in any language were eligible for inclusion.

### Information sources

Literature searches were conducted in following bibliographic databases: MEDLINE, Embase, PsycINFO, The Cochrane Library (Cochrane Database of Systematic Reviews (CDRS), the Cochrane Central Register of Controlled Trials (CENTRAL), Database of Abstracts of Reviews of Effects (DARE)), Scopus, and Epistemonikos. Additionally, ClinicalTrials.gov was searched for unpublished trials that were relevant for this study. The databases were searched from 01 January 1994 to 30 November 2018. To find relevant studies potentially ignored by the database searches, reference lists of relevant publications were also manually screened.

### Search strategy

The strategy was tested, revised, and finalized by a member of the team with a medical librarian background (SAP). The search included thesaurus- and free-text terms optimized to identify bibliographic references involving ASD and OCD. Hedges optimized to identify randomized controlled trials in MEDLINE [26], Embase [27] and PsycInfo [28], were used to restrict the search to this study type. A detail description of the search strategy for the different databases is available as Additional file 2.

### Study selection

All references from the literature search were imported to Endnote X7.2. Duplicates were removed. Two reviewers screened the titles and abstracts of all records identified by the search, first independently and then jointly. Records were promoted to full text screening if they met the following pre-specified eligibility criteria: (1) RCT comparing medications with placebo; (2) Medical treatment of restricted /repetitive behavior or interests, including compulsive behaviors and stereotypies on ASD patients of any gender and age; (3) The outcome measures used were RBS-Revised, ABC, and/or YBOCS and its child version CY-BOCS. Two reviewers (A.C., Y.Y) assessed all the relevant studies in full text, independently, and then jointly, based on pre-specified eligibility criteria. Reasons for exclusion recorded and when there were any doubts about inclusion of a study, these were also documented, and the doubts were resolved by discussion with the collaborators (N.S, B.L, B.A.) with expertise in ASD and Pervasive Developmental Disorder. Study investigators listed in ClinicalTrials.gov were contacted to retrieve information about unpublished studies of relevance.

### Data collection process

Two reviewers (A.C, Y.Y) extracted the relevant data from the included studies with a predefined data recording form, which addressing search criteria (Details are provided as Additional file 1). Both reviewers then checked the completeness and accuracy of the data extraction for all included studies. To resolve discrepancies, collaborators (N.S, B.L, B.A) were consulted to help with consensus development. The following core data were extracted from all included studies: Title, authors, and other publication details; Study design and aim, Setting (place and time of recruitment/data collection), Study Population characteristics (age, gender, and diagnostic criteria used, sample size etc.) Intervention characteristics (type of medication used, duration of intervention); Methods of outcome measurement (ABC, RBS-Revised-BOCS, CY-BOCS); Statistical methods and results related to the outcomes (Mean, Standard deviation, the only data which could be extracted from the original papers for meta-analysis).

### Risk of bias in individual studies

Two reviewers (A.C, Y.Y) assessed Risk of Bias (RoB), independently and then jointly.RoB was assessed for each included RCT in accordance with the criteria in the Cochrane Handbook for Systematic Reviews of Interventions [29]. The following key domains were used to assess RoB: (a) sequence generation; (b) allocation concealment; (c) blinding of participants and personnel; (d) blinding of outcome assessment; (e) incomplete outcome data; (f) selective outcome reporting; and, (g) other sources of bias. Response options of ‘Low Risk’, ‘Unclear Risk’, and ‘High Risk’ for each of the domains were documented. Studies were assigned as low risk of bias across each domain if no potential source of bias were found. In case of lack of information, or uncertainty over the potential source of bias, the studies were assigned as having unclear risk of bias. Similarly, if a potential source of bias was found, the studies were assigned as high risk of bias across the aforementioned domain in the included studies [29]. Any disagreements between the two reviewers were resolved by discussion with the collaborators.

### Synthesis of results

Data were summarized and presented narratively in text and tables for each comparison. As the outcome variables were continuous, the group post-test means and standard deviations were used to calculate effect sizes, using Review Manager 5.3 (RevMan 2014). Since the included studies used different scales to measure the same outcome, standardized mean differences (SMD) with corresponding 95% CI’s were calculated to estimate effect size. RevMan 2014 was then used to pool the data (meta-analyses) and generate forest plots to display the results. Publication bias was assessed by plotting the effect size against standard error for each trial using a funnel plot.

## Results

### Study selection

**Fig. 1:**
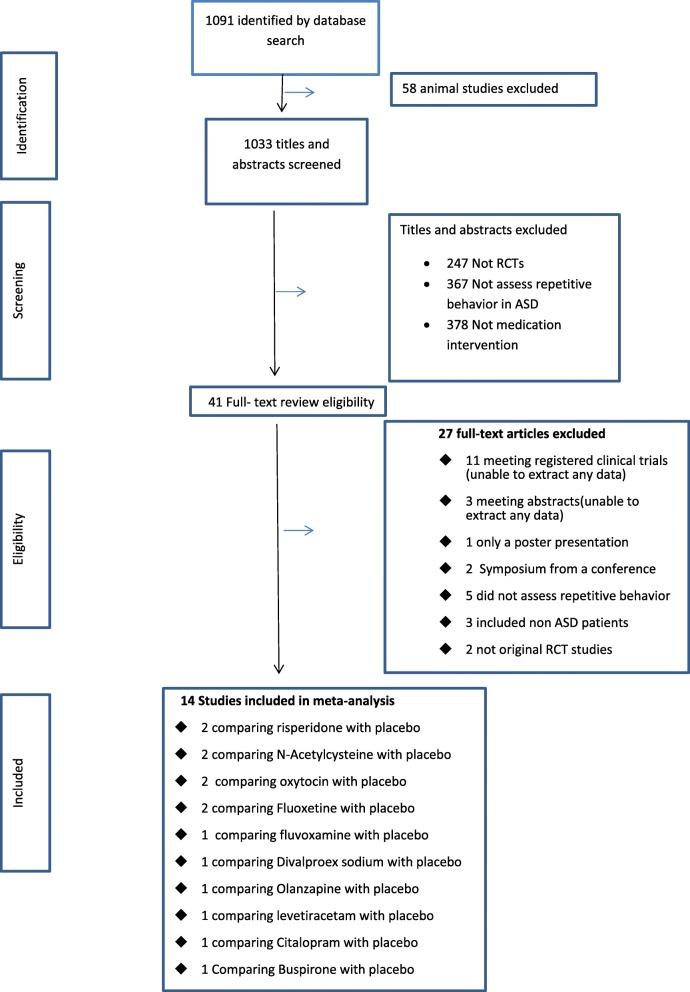
Flow diagram

In total, 1091 unique references were identified by the literature search. After screening the title and abstracts, 41 relevant studies selected and subjected to full-text review (Fig. 1). Among them, 27 papers were excluded because of: 1) Target individuals were not solely ASD (i.e. comorbidity); 2) the intervention did not assess repetitive behavior; 3) studies had a non-randomized design; 4) full text articles were not available and, 5) 11 clinical trials without available data, 4 authors with possible ways to contact were approached by email and telephone, and none of them reply.

Fourteen RCTs comparing efficacy of 10 medications with placebo, published in English between 1994 and 2018, were identified as eligible (Fig. 1 and Table 1). For the meta-analysis, nine of 14 studies [30–32, 36–39, 41, 42] with data on mean and standard deviation were included.

**Table 1:** A summary of the 14 including RCT studies with medication interventions assessing the restricted/repetitive behavior in autism spectrum disorders patients

Study	Participants (Age range)	Participants sex distribution	Participants Race	Sample size	Diagnostic criteria	Intervention	Comparison	Intervention vs placebo	Assessment scales	Result	Included in metaanalysis
McDougle et. al., 1996 [30]	18- 53	27 male /3 female	Not reported	30	ICD10	fluvoxamine 15 (50 - 300mg/day)	Placebo	15vs15	Y-BOCS	positive	Included
McDougle et.al.,1998 [31]	18-43	22 male/9 female	6 African Americans, 24 whites, and 1 Hispanic	31	DSM-IV-TR	risperidone,15 (1-10 mg/day)	Placebo	15vs16	Y-BOCS	positive	Included
Hollander et. al., 2005 [32]	5–16	30 male -9 female	57% of the subjects were Caucasian, 23% Black, 15% Hispanic and 5% Asian	39	DSM-IV-TR	Fluoxetine,20 (0.8 mg/kg/day)	Placebo	20vs19	Y-BOCS	negative	Included
Hollander et.al., 2006 [33]	5-17 (one is 40)	Not reported	Eight of the subjects were Caucasian, two were African American, two were Asian and one was Hispanic	13	DSM-IV-TR	Divalproex sodium,9 (125 mg/d)	Placebo	9vs4	CY-BOCS	positive	
Wasserman et.al., 2006 [34]	5–17	17 male /3 female	50% Caucasian, 35% African American, 5% Asian, and 10% Hispanic.	20	DSM-IV-TR	levetiracetam, 10 (125 - 250mg/day)	Placebo	10vs10	CY-BOCS	negative	
Hollander et. al., 2006 [35]	14-6	9 male /2 female	7 Caucasian, 1 Hispanic, 1 Asian, and 2 African American	11	DSM-IV-TR	Olanzapine,6 (2.5-20 mg/day)	Placebo	6vs5	CY-BOCS	negative	
King et. al., 2009 [36]	17-5	128 male/21 female	White 108 Black 17 Asian 14 Other 11	149	DSM-IV-TR	Citalopram,73 2.5-20mg/d.)	Placebo	73vs76	CY-BOCS	negative	Included
Anagnostou. et. al., 2012 [37]	mean(33.2 ±13.3)	16 male /3 female	Caucasian 14 Black 1 Hispanic 1 Asian 1 other 1	19	DSM-IV-TR	Oxytocin,10 (4 8IU/d)	Placebo	10vs9	Y-BOCS&RBS-R	RBS-R low prder 0.045	Included
Hardan et. al., 2012 [38]	3.2–10.7	2 male /31 female	not clear	33	DSM-IV-TR	NAcetylcysteine, 15 (900-2700 mg/day)	Placebo	15vs18	Y-BOCS&RBS-R	RBS-R stereotypies 0.014	Included
Hollander et al., 2012 [39]	18-60	26 male /11 female	57% of the subjects were Caucasian, 23% Black, 15% Hispanic and 5% Asian	37	DSM-IV-TR	fluoxetine, 22 (10-80mg/day)	Placebo	22vs15	CY-BOCS	positive	Included
Kent et. al.,2012 [40]	5 -17	12 male /84 female	White 67 Black 19 Asian 7 Other 3	96	DSM-IV-TR	Risperidone (0.125 mg/day [20 to\45 kg], 0.175 mg/day [[45 kg]), 20 Risperidone (high dose1.25 mg/day [20 to \45 kg], 1.75 mg/day [[45 kg])),31	Placebo	20vs31vs35	ABC&CYBOCS	positive	
Diane C. Chugani et al. 2016 [41]	2 -<6	137 male/29 female	American Indian or Alaskan 1 Asian 2 black 51 hawaiian white 97 other 12	166	DSM-IV-TR	Buspirone (2.5 mg/Ml, low dose),54 Buspirone (5 mg/Ml, high dose),55	Placebo	54vs55vs57	Y-BOCS&RBS	positive	Included
Dean et al., 2017 [42]	3-9	79 male/19 female	Australia and Anglo-Saxon	102	DSM-IV-TR	N-acetyl cysteine,51 (500 - 2000mg/day)	Placebo	51vs51	RBS-R	negative	Included
Parker et. al., 2017 [43]	6–12	27 male /5 female	Caucasian Asian	32	DSM-IV-TR	oxytocin, 14 (18-24IU/d)	Placebo	14vs18	RBS-R	negative	Included

*ASD* autism spectrum disorder, *DSM-IV-TR* Diagnostic and Statistical Manual IV text revision, *ICD-10* International Classification of Disease, Tenth Revision, *RBS-R* Repetitive Behavior Scale - Revised, *Y-BOCS* Yale–Brown Obsessive–Compulsive Scale, *CY-BOCS* Children Yale–Brown Obsessive–Compulsive Scale

### Study characteristics

The 14 selected studies included 778 individuals. The mean study sample size was 55.6, ranging from 11 to 166. Overall, 348 participants were randomly assigned to medication and 430 to placebo. Most of the sample population were male (633 of 765). The mean age of study participants varied considerably: four studies evaluated treatment for adults (range 18–60) [30, 31, 37, 39], while 10 involved evaluated interventions only for children and adolescents (range 2–18) [32–36, 38–43]. The median duration of the treatment period was 12 weeks (range 6–24). Ten (72.4%) trials recruited patients with varying ethnicity, including African, European, Asian, and Hispanic (Table 1).

### Methodological quality of included studies

**Fig. 2:**
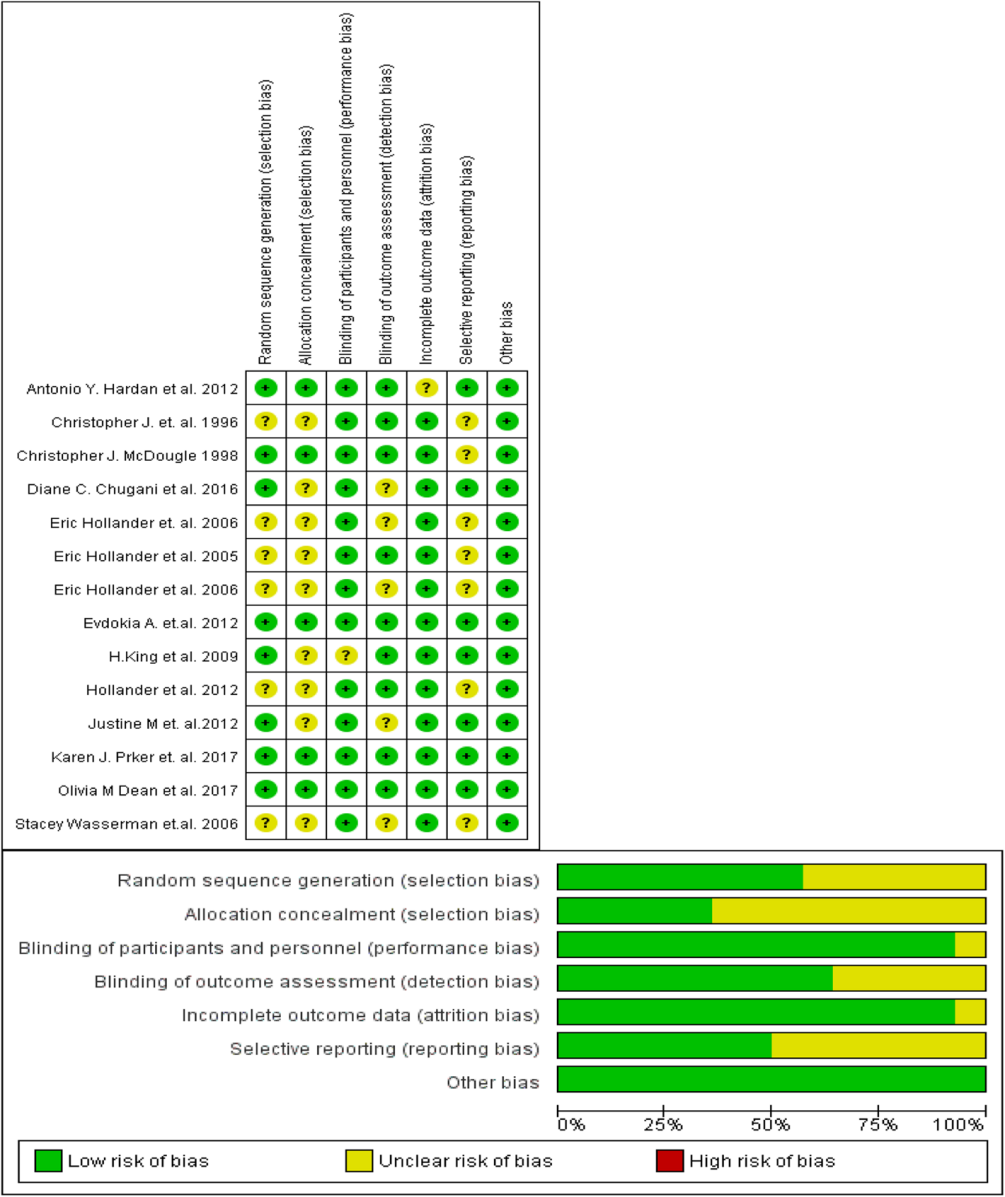
RoB graph & RoB summary: RoB graph review authors’ judgements about each RoB item presented as percentages across all included studies. RoB summary: review authors’ judgements about each RoB item for each included study

Risk of bias (RoB) assessment for the included studies found low or unclear risk across different domains of assessment, including Selection bias, Performance bias, Detection bias, Attrition bias, Reporting bias and others. The RoB assessment, with the judgment and the explanation supporting the judgment for each domains, is summarized in Fig. 2.

### Clinical outcome

Among the 14 RCT studies found eligible, six found significant improvement as assessed by RRBs scales when comparing medication and placebo groups: two on risperidone [31, 40]; one on fluvoxamine [30]; one on fluoxetine [39]; one on Buspirone [41] and, one divalproex sodium [33]. Six studies yielded negative results: one on n-acetylcysteine [42]; one on oxytocin [43]; one on fluoxetine [32]; one on citalopram [36]; one levetiracetam [34]; and, one olanzapine [35]). Two studies showed a significant difference on only one of the two subscales of the RBS-R (stereotypies) of the two tools (RBS-R and Y-BOCS) used in the study: one oxytocin [31]; one N-Acetylcysteine [38] (See Table 1).

### Synthesis of meta-analyses

**Fig. 3:**
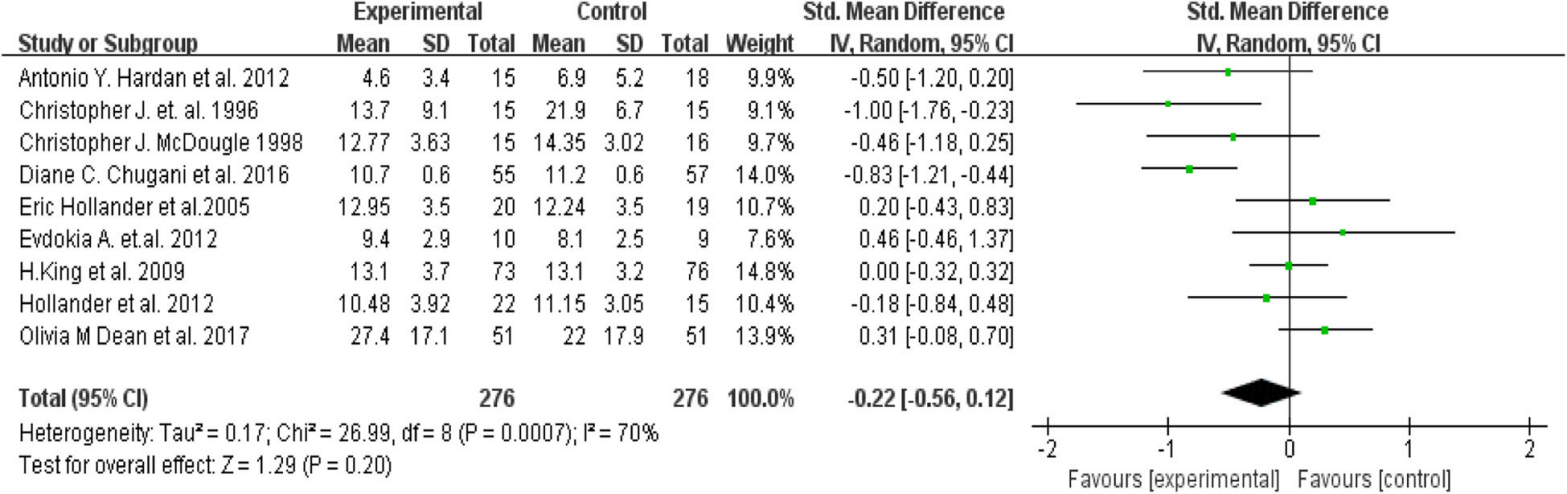
Forest plot comparing efficacy of pharmacotherapy over placebo in reducing repetitive behaviour in patient with autism

Nine of the fourteen [30–32, 36–39, 41, 42] included studies provided outcomes in terms of means and standard deviations of RRB assessment scale scores; these 9 were included in the meta-analysis (Fig. 3). The random effects meta-analysis identified no significant differences between medication group and placebo group in any of the nine studies included (Tau^2^ = 0.17; Chi^2^ = 26.99, z = 1.29, *p* = 0.20) (Fig. 3). Visual inspection of the I^2^ statistic in the forest plot indicates the presence of significant heterogeneity between these studies [df = 8, *P* = 0.0007, I^2^ = 70%].

**Fig. 4:**
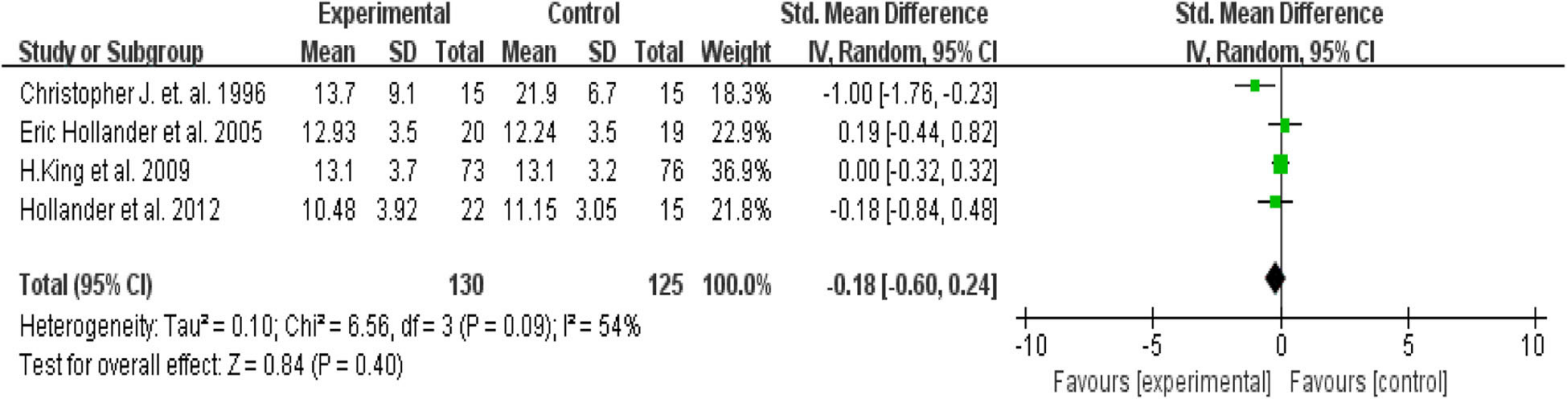
Forest plot comparing efficacy of SSRIs over placebo in reducing repetitive behaviour in patient with autism

**Fig. 5:**
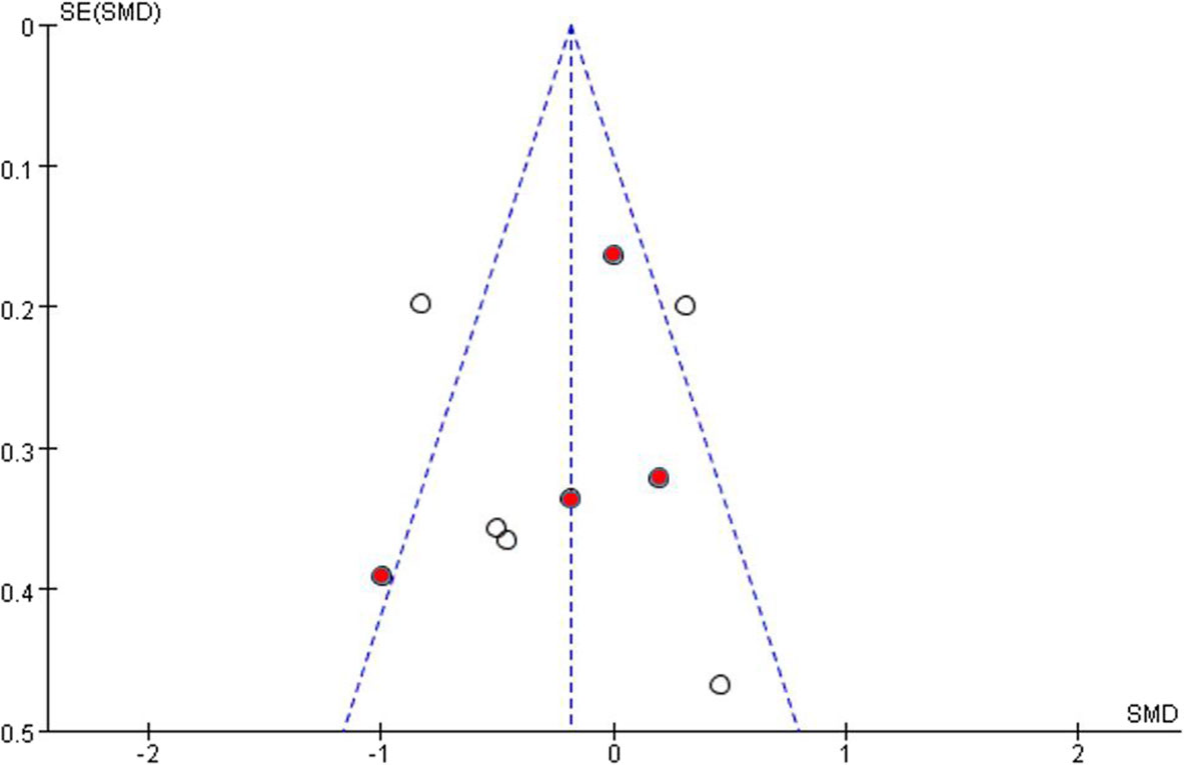
Funnel plot comparing publication bias of included papers(Red dots indicate SSRI studies)

In the sub-group analysis between the selective serotonin reuptake inhibitor (SSRI) and placebo (four studies) no significant difference was found between medication and placebo groups when using the RRB assessment scales (Tau^2^ = 0.10; Chi^2^ = 6.59, Z = 0.83, *P* = 0.41) (Fig. 4). On visual inspection of the forest plot, I^2^ statistic also identified the presence of relatively significant heterogeneity [df = 3, *P* = 0.09, I^2^ = 54%]. A funnel plot assessing publication bias among the included papers reveals that studies included in the meta-analysis were symmetrically scattered about the mean effect size, indicating no publication bias. (Fig. 5).

## Discussion

This study updates the present knowledge on the effectiveness of pharmacotherapy in treating restricted/repetitive behavior in ASD, based on randomized control trials published in the period 1994–2018.

Among the 14 included studies, six showed a significant difference between active medication, risperidone [31, 40]; fluvoxamine [30]; fluoxetine [39]; Buspirone [41]; divalproex sodium [33], and placebo in reducing RRBs. While these individual studies report that pharmacological treatment may be effective against RRBs among patients with ASD, our meta-analysis showed no significant differences between medication and placebo, neither when including all intervention (552 patients) nor when restricting to only SSRI intervention (255 patients). The results offer further evidence suggesting that SSRIs have little or no effect on treatment of RRBs in children and adults patients with ASD [44]. A previous meta-analysis showed a small, but significant, effect of SSRIs in treating repetitive behavior in ASD, but also indicated that after adjusting for publication bias, the result was insignificant [18]. A sub-group meta-analysis from a similar study also found no significant differences between SSRIs and placebo [16]. Another review indicated that serotonin reuptake inhibitors (SRIs) are less effective and less tolerated in children with autism compared to adults [45]. A review by Doyle and McDougle suggesting that SRIs are more effective in adults and older adolescents compared to children for the treatment of repetitive behavior, and that children may exhibit more behavioral activation from this type of treatment [46]. Hence, the findings from the earlier studies are consistent with our findings.

The findings of this meta-analysis should be interpreted with some caution due to the limitations:

- First, there exists high level of heterogeneity between studies, due to clinical diversity (e.g., different age and ancestry; different intervention), methodological diversity (different sample size, dosing, treatment period, methods of RRB measurement) and statistical diversity (different risk of bias).
- Second, the presence of significant heterogeneity in meta-analysis results indicates that even though the results showed no significant effect, there may still be an effect, thus creating the possibility of a Type II error.
- Third, only studies with available data on means and standard deviations of RRBs were included in meta-analysis. This omitted two positive studies [32, 40] from our analysis.
- Fourth, several unpublished clinical trials corresponded to our inclusion criteria; however, they were excluded from meta -analysis because data were unavailable.
- Fifth, it is also possible that our search strategy (e.g., publication from 1994 to 2018) might have limited our studies.
- Sixth, we did not analyze data on medication side effects because only a few studies assessed side effect therefore we do not have enough data for analysis.
- Seventh, all our included papers compared drugs with placebo, which result in our study to be indirect treatment comparison, and our result to be indirect evidences for medication using. Network meta-analysis is considered a good way to deal with indirect treatment comparison. However, the lack of data limited our ability to do it. Future study is recommended to do network meta-analysis if possible.

All studies included in our analysis were RCTs that could provide rigorous evidence of the effectiveness of medication for reducing the RRBs symptoms in individuals with ASD. Previous pharmacological research has targeted interfering symptom domains associated with ASD; these domains include hyperactivity and inattention, irritability, core social impairment and RRBs. However, most studies focus on the disorder as a whole [20, 47, 48], while only few studies have been focused on pharmacotherapy against RRBs symptoms in ASD specifically.

## Conclusions

Pharmacotherapy studies in ASD contribute to our understanding the etiological substrates of ASD as well as the efficacy of medication in treatment of core symptoms of ASD. However, methodological variability, such as different study design, sample size, different dosage and treatment duration complicates inter-study comparisons, making it difficult to determine the efficacy of available medications. And what important is how to build reliable and sensitive outcome measures to collect behavioural symptom need to be considered more, in addition double-blind, placebo-controlled trials for RRB’s in ASD were still not enough. These constitute big challenges in our study on efficacy of pharmacological treatment of RRB’s in ASD patients.

Significant progress has been made in the development of effective drug treatment against irritability. A similar development with regards to reducing RRB’s in patients with ASD should also be a high priority as these symptoms severely disrupt adaptation and interfere with crucial support systems for individuals with ASD and their families. Our review reveals paucity of large balanced trials with precise assessment tools and long-term follow-up targeting RRB in ASD patients. Additional studies of this type are required to make the necessary progress in this area.

## Supplementary information

**Additional file 1.** PRISMA checklist.

**Additional file 2.** Detail search strategy.

## Data Availability

All data generated or analysed during this study are included in this published article [and its supplementary information files].

## Abbreviations

AC: Ashmita Chaulagain
BA: Branko Aleksic
BL: Bennett L. Leventhal
NK: Norbert Skokauskas
NO: Norio Ozaki
PZ: Peter Szatmari
SL: Stian Lydersen
SP: Sindre Andre Pedersen
YY: Yanjie Yu
