# Supplementary material for: Pharmacotherapy of restricted/repetitive behavior in autism spectrum disorder:a systematic review and meta-analysis
Source: BMC Psychiatry. 2020 Mar 12;20:121. doi: 10.1186/s12888-020-2477-9 (PMC7068977; doi:10.1186/s12888-020-2477-9)
Supplement: Supplementary file 2 — Additional file 2. Detail search strategy. [file 12888_2020_2477_MOESM2_ESM.docx]

**Additional file 1**

**Pharmacotherapy of restricted/repetitive behavior in Autism Spectrum Disorder:**

**A systematic review and meta analysis**

Yanjie Yu, Ashmita Chaulagain, Sindre Andre Pedersen, Stian Lydersen, Bennett L. Leventhal, Peter Szatmari,Branko Aleksic, Norio Ozaki, Norbert Skokauskas

***Contents of Supplementary Materials***

**Details of searching strategy**

**MEDLINE**:

|  | | | | | | |
| --- | --- | --- | --- | --- | --- | --- |
| 1 | exp Child Development Disorders, Pervasive/ |  |  |  |  |  |
| 2 | exp Autism Spectrum Disorder/ |  |  |  |  |  |
| 3 | exp Autistic Disorder/ |  |  |  |  |  |
| 4 | autism*.ti,ab,kw. |  |  |  |  |  |
| 5 | autistic.ti,ab,kw. |  |  |  |  |  |
| 6 | ASD.ti,ab,kw. |  |  |  |  |  |
| 7 | asperger*.ti,ab,kw. |  |  |  |  |  |
| 8 | kanner*.ti,ab,kw. |  |  |  |  |  |
| 9 | pervasive.ti,ab,kw. |  |  |  |  |  |
| 10 | "childhood disintegrative disorder*".ti,ab,kw. |  |  |  |  |  |
| 11 | CDD.ti,ab,kw. |  |  |  |  |  |
| 12 | heller*.ti,ab,kw. |  |  |  |  |  |
| 13 | rett*.ti,ab,kw. |  |  |  |  |  |
| 14 | (disintegrative adj3 (psychosis or disorder*)).ti,ab,kw. |  |  |  |  |  |
| 15 | (child* adj3 schizophren*).ti,ab,kw. |  |  |  |  |  |
| 16 | ((intellectual or mental) adj3 (disab* or retard* or handicap*)).ti,ab,kw. |  |  |  |  |  |
| 17 | or/1-16 |  |  |  |  |  |
| 18 | exp Obsessive-Compulsive Disorder/ |  |  |  |  |  |
| 19 | exp Hoarding Disorder/ |  |  |  |  |  |
| 20 | OCD.ti,ab,kw. |  |  |  |  |  |
| 21 | obsess*.ti,ab,kw. |  |  |  |  |  |
| 22 | compuls*.ti,ab,kw. |  |  |  |  |  |
| 23 | hoarding.ti,ab,kw. |  |  |  |  |  |
| 24 | ritual*.ti,ab,kw. |  |  |  |  |  |
| 25 | repetitive.ti,ab,kw. |  |  |  |  |  |
| 26 | "preoccupation neurosis".ti,ab,kw. |  |  |  |  |  |
| 27 | "anakastric personalit*".ti,ab,kw. |  |  |  |  |  |
| 28 | or/18-27 |  |  |  |  |  |
| 29 | randomized controlled trial.pt. |  |  |  |  |  |
| 30 | randomized.mp. |  |  |  |  |  |
| 31 | placebo.mp. |  |  |  |  |  |
| 32 | or/29-31 |  |  |  |  |  |
| 33 | 17 and 28 and 32 |  |  |  |  |  |
|  | | |  |  | | |

**Embase:**

| 1 | exp autism/ |
| --- | --- |
| 2 | autism*.ti,ab,kw. |
| 3 | autistic.ti,ab,kw. |
| 4 | ASD.ti,ab,kw. |
| 5 | asperger*.ti,ab,kw. |
| 6 | kanner*.ti,ab,kw. |
| 7 | pervasive.ti,ab,kw. |
| 8 | "childhood disintegrative disorder*".ti,ab,kw. |
| 9 | CDD.ti,ab,kw. |
| 10 | heller*.ti,ab,kw. |
| 11 | rett*.ti,ab,kw. |
| 12 | (disintegrative adj3 (psychosis or disorder*)).ti,ab,kw. |
| 13 | (child* adj3 schizophren*).ti,ab,kw. |
| 14 | ((intellectual or mental) adj3 (disab* or retard* or handicap*)).ti,ab,kw. |
| 15 | or/1-14 |
| 16 | exp Obsessive Compulsive Disorder/ |
| 17 | exp Hoarding/ |
| 18 | exp Compulsion/ |
| 19 | exp Obsession/ |
| 20 | OCD.ti,ab,kw. |
| 21 | obsess*.ti,ab,kw. |
| 22 | compuls*.ti,ab,kw. |
| 23 | hoarding.ti,ab,kw. |
| 24 | ritual*.ti,ab,kw. |
| 25 | repetitive.ti,ab,kw. |
| 26 | "preoccupation neurosis".ti,ab,kw. |
| 27 | "anakastric personalit*".ti,ab,kw. |
| 28 | or/16-27 |
| 29 | random*.tw. |
| 30 | placebo*.mp. |
| 31 | double-blind*.tw. |
| 32 | or/29-31 |
| 33 | 15 and 28 and 32 |

**PsycINFO:**

| 1 | exp Autism Spectrum Disorders/ |  |  |  |  |  |
| --- | --- | --- | --- | --- | --- | --- |
| 2 | autism*.tw. |  |  |  |  |  |
| 3 | autistic.tw. |  |  |  |  |  |
| 4 | ASD.tw. |  |  |  |  |  |
| 5 | asperger*.tw. |  |  |  |  |  |
| 6 | kanner*.tw. |  |  |  |  |  |
| 7 | pervasive.tw. |  |  |  |  |  |
| 8 | "childhood disintegrative disorder*".tw. |  |  |  |  |  |
| 9 | CDD.tw. |  |  |  |  |  |
| 10 | heller*.tw. |  |  |  |  |  |
| 11 | rett*.tw. |  |  |  |  |  |
| 12 | (disintegrative adj3 (psychosis or disorder*)).tw. |  |  |  |  |  |
| 13 | (child* adj3 schizophren*).tw. |  |  |  |  |  |
| 14 | ((intellectual or mental) adj3 (disab* or retard* or handicap*)).tw. |  |  |  |  |  |
| 15 | or/1-14 |  |  |  |  |  |
| 16 | exp obsessive compulsive disorder/ |  |  |  |  |  |
| 17 | OCD.tw. |  |  |  |  |  |
| 18 | obsess*.tw. |  |  |  |  |  |
| 19 | compuls*.tw. |  |  |  |  |  |
| 20 | hoarding.tw. |  |  |  |  |  |
| 21 | ritual*.tw. |  |  |  |  |  |
| 22 | repetitive.tw. |  |  |  |  |  |
| 23 | "preoccupation neurosis".tw. |  |  |  |  |  |
| 24 | "anakastric personalit*".tw. |  |  |  |  |  |
| 25 | or/16-24 |  |  |  |  |  |
| 26 | double-blind.tw. |  |  |  |  |  |
| 27 | random* assigned.tw. |  |  |  |  |  |
| 28 | control.tw. |  |  |  |  |  |
| 29 | 15 and 25 and 28 |  |  |  |  |  |
|  | | |  |  | | |

**The Cochrane Library:**

ID Search Hits

#1 MeSH descriptor: [Child Development Disorders, Pervasive] explode all trees

#2 MeSH descriptor: [Autism Spectrum Disorder] explode all trees

#3 MeSH descriptor: [Autistic Disorder] explode all trees

#4 autism*:ti,ab,kw (Word variations have been searched)

#5 autistic:ti,ab,kw (Word variations have been searched)

#6 ASD:ti,ab,kw (Word variations have been searched)

#7 asperger*:ti,ab,kw (Word variations have been searched)

#8 kanner*:ti,ab,kw (Word variations have been searched)

#9 pervasive:ti,ab,kw (Word variations have been searched)

#10 "childhood disintegrative disorder*":ti,ab,kw (Word variations have been searched)

#11 CDD:ti,ab,kw (Word variations have been searched)

#12 heller*:ti,ab,kw (Word variations have been searched)

#13 rett*:ti,ab,kw (Word variations have been searched)

#14 disintegrative near/3 (psychosis or disorder*):ti,ab,kw (Word variations have been searched)

#15 child* near/3 schizophren*:ti,ab,kw (Word variations have been searched) 104

#16 (intellectual or mental) near/3 (disab* or retard* or handicap*):ti,ab,kw (Word variations have been searched)

#17 #1 or #2 or #3 or #4 or #5 or #6 or #7 or #8 or #9 or #10 or #11 or #12 or #13 or #14 or #15 or #16

#18 MeSH descriptor: [Obsessive-Compulsive Disorder] explode all trees

#19 MeSH descriptor: [Hoarding Disorder] explode all trees

#20 OCD:ti,ab,kw (Word variations have been searched)

#21 obsess*:ti,ab,kw (Word variations have been searched)

#22 compuls*:ti,ab,kw (Word variations have been searched)

#23 hoarding:ti,ab,kw (Word variations have been searched)

#24 ritual*:ti,ab,kw (Word variations have been searched)

#25 repetitive:ti,ab,kw (Word variations have been searched)

#26 "preoccupation neurosis":ti,ab,kw (Word variations have been searched)

#27 "anakastric personalit*":ti,ab,kw (Word variations have been searched)

#28 #18 or #19 or #20 or #21 or #22 or #23 or #24 or #25 or #26 or #27

#29 #17 and #28

**Scopus:**

( ( TITLE-ABS-KEY ( autism* ) ) OR ( TITLE-ABS-KEY ( autistic ) ) OR ( TITLE-ABS-KEY ( asd ) ) OR ( TITLE-ABS-KEY ( asperger* ) ) OR ( TITLE-ABS-KEY ( kanner* ) ) OR ( TITLE-ABS-KEY ( pervasive ) ) OR ( TITLE-ABS-KEY ( "childhood disintegrative disorder*" ) ) OR ( TITLE-ABS-KEY ( cdd ) ) OR ( TITLE-ABS-KEY ( heller* ) ) OR ( TITLE-ABS-KEY ( rett* ) ) OR ( TITLE-ABS-KEY ( disintegrative W/3 ( psychosis OR disorder* ) ) ) OR ( TITLE-ABS-KEY ( child* W/3 schizophren* ) ) OR ( TITLE-ABS-KEY ( ( intellectual OR mental ) W/3 ( disab* OR retard* OR handicap* ) ) ) ) AND ( ( TITLE-ABS-KEY ( ocd ) ) OR ( TITLE-ABS-KEY ( obsess* ) ) OR ( TITLE-ABS-KEY ( compuls* ) ) OR ( TITLE-ABS-KEY ( hoarding ) ) OR ( TITLE-ABS-KEY ( ritual* ) ) OR ( TITLE-ABS-KEY ( repetitive ) ) OR ( TITLE-ABS-KEY ( "preoccupation neurosis" ) ) OR ( TITLE-ABS-KEY ( "anakastric personalit*" ) ) ) AND ( ( INDEXTERMS ( "clinical trials" OR "clinical trials as a topic" OR "randomized controlled trial" OR "Randomized Controlled Trials as Topic" OR "controlled clinical trial" OR "Controlled Clinical Trials" OR "random allocation" OR "Double-Blind Method" OR "Single-Blind Method" OR "Cross-Over Studies" OR "Placebos" OR "multicenter study" OR "double blind procedure" OR "single blind procedure" OR "crossover procedure" OR "clinical trial" OR "controlled study" OR "randomization" OR "placebo" ) ) OR ( TITLE-ABS-KEY ( ( "clinical trials" OR "clinical trials as a topic" OR "randomized controlled trial" OR "Randomized Controlled Trials as Topic" OR "controlled clinical trial" OR "Controlled Clinical Trials as Topic" OR "random allocation" OR "randomly allocated" OR "allocated randomly" OR "Double-Blind Method" OR "Single-Blind Method" OR "Cross-Over Studies" OR "Placebos" OR "cross-over trial" OR "single blind" OR "double blind" OR "factorial design" OR "factorial trial" ) ) ) OR ( TITLE-ABS ( clinical AND trial* OR trial* OR rct* OR random* OR blind* ) ) ) AND NOT (INDEX ( medline ) OR INDEX ( embase ))

**Epistimonikos:**

(title:((Autism* OR autistic OR ASD OR asperger* OR kanner* OR pervasive OR disintegrative OR CDD OR heller* OR rett* OR (child* AND schizophren*)) AND (OCD OR obsess* OR compuls* OR hoarding OR ritual* OR repetitive)) OR abstract:((Autism* OR autistic OR ASD OR asperger* OR kanner* OR pervasive OR disintegrative OR CDD OR heller* OR rett* OR (child* AND schizophren*)) AND (OCD OR obsess* OR compuls* OR hoarding OR ritual* OR repetitive))) Publication year: 2011-2019

**ClinicalTrials.gov:**

(Obsessive compulsive disorder OR "obsessive compulsive" OR "obsessive-compulsive" OR OCD OR Repetitive Behaviors OR "repetitive behavior" OR "repetitive behaviour") AND Autism Spectrum Disorder
